# The new French 2010 *Rabbit Hemorrhagic Disease Virus* causes an RHD-like disease in the Sardinian Cape hare (*Lepus capensis mediterraneus*)

**DOI:** 10.1186/1297-9716-44-96

**Published:** 2013-10-07

**Authors:** Giantonella Puggioni, Patrizia Cavadini, Caterina Maestrale, Rosario Scivoli, Giuliana Botti, Ciriaco Ligios, Ghislaine Le Gall-Reculé, Antonio Lavazza, Lorenzo Capucci

**Affiliations:** 1 Istituto Zooprofilattico Sperimentale della Sardegna, via Duca degli Abruzzi 8, 07100 Sassari, Italy; 2 Istituto Zooprofilattico Sperimentale della Lombardia ed Emilia Romagna, OIE Reference Laboratory for RHD, via Bianchi 9, 25124 Brescia, Italy; 3 Anses, French Agency for Food, Environmental and Occupational Health & Safety, Ploufragan-Plouzané Laboratory Avian and Rabbit Virology, Immunology and Parasitology Unit, BP 53, 22440 Ploufragan, France; 4 European University of Brittany, 5 Boulevard Laennec, 35000 Rennes, France

## Abstract

*Lagovirus* is an emerging genus of *Caliciviridae*, which includes the *Rabbit Hemorrhagic Disease Virus* (RHDV) of rabbits and the *European brown hare syndrome virus* (EBHSV) of hares that cause lethal hepatitis. In 2010, a new RHDV related virus (RHDV2) with a unique genetic and antigenic profile and lower virulence was identified in France in rabbits. Here we report the identification of RHDV2 as the cause in Sardinia of several outbreaks of acute hepatitis in rabbits and Cape hare (*Lepus capensis mediterraneus*). This is the first account of a lagovirus that causes fatal hepatitis in both rabbits and hares.

## Introduction, methods and results

Rabbit Hemorrhagic Disease (RHD) and European Brown Hare Syndrome (EBHS) are two similar acute and lethal forms of hepatitis caused by two distinct species-specific lagoviruses that emerged around 1980 in China and in northern Europe, respectively [1-3]. Since then, other virulent RHDV antigenic variants [4,5] and especially non-pathogenic related viruses, such as rabbit calicivirus (RCV), have been increasingly detected [6-9]. In 2010 a new RHDV related virus with a discrete genetic profile and distinctive phenotypic traits, was identified in France [10]. Further studies showed it also has a specific antigenic profile and an average mortality of 20% in experimentally infected rabbits, which is consistently less than RHDV [11]. The data available suggest that this virus, which has the proposed name RHDV2, is not a variant of RHDV but a newly emerged virus [11]. RHDV2 is now spreading in Europe as demonstrated by its detection both in Italy [11,12] and Spain during 2011 [13,14].

**Figure 1:**
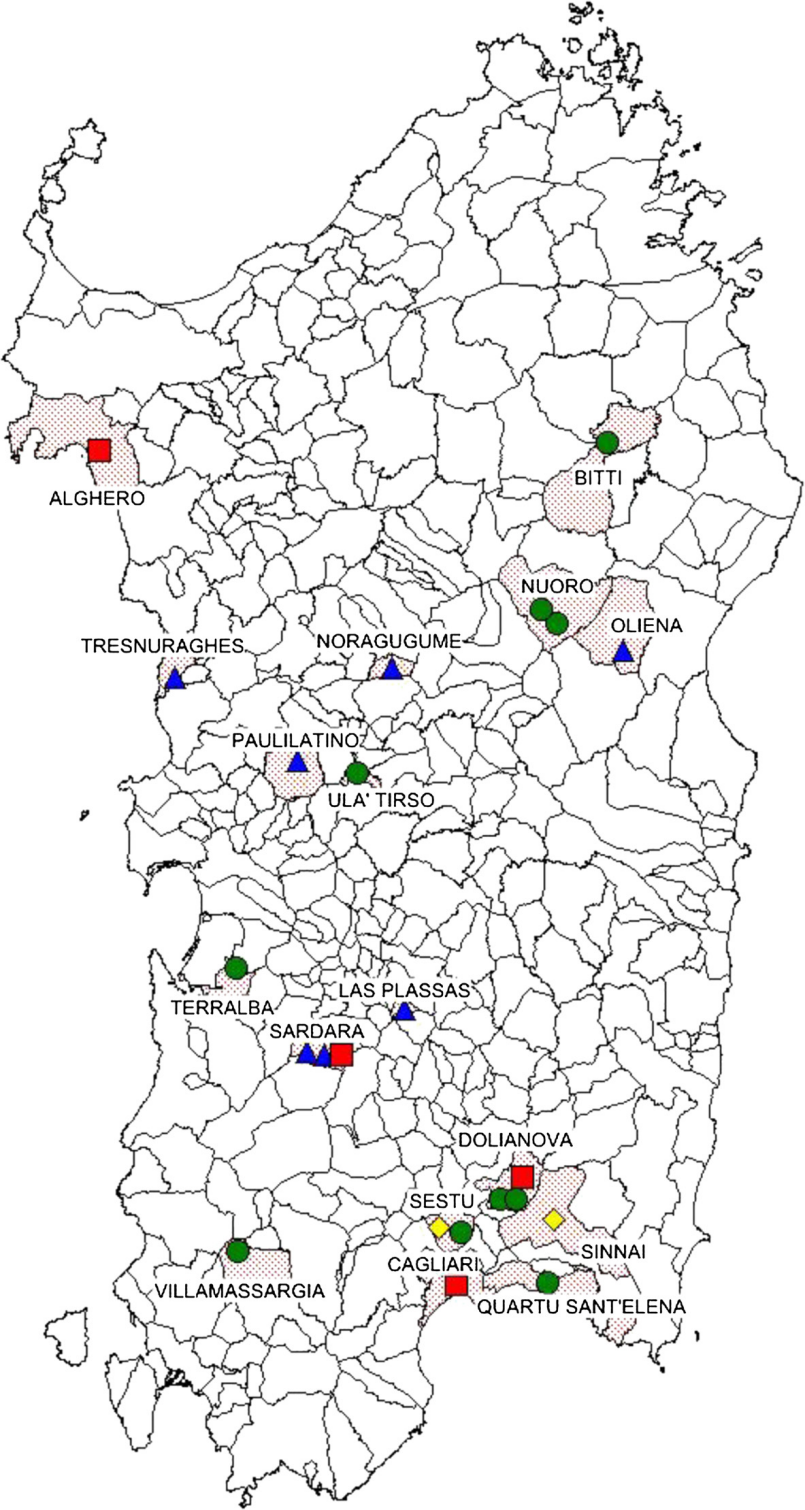
**Geographic range of rabbit hemorrhagic disease (RHD) outbreaks in rabbits and Cape hares (*Lepus capensis mediterraneus *) in Sardinia from 2008 to 2012.** Green = rabbits infected by RHDV during 2008–2010; yellow: rabbits infected by RHDV in 2011; red: rabbits infected by RHDV2 during 2011–2012; blue: Cape hares infected by RHDV2 in the autumn of 2011.

**Figure 2:**
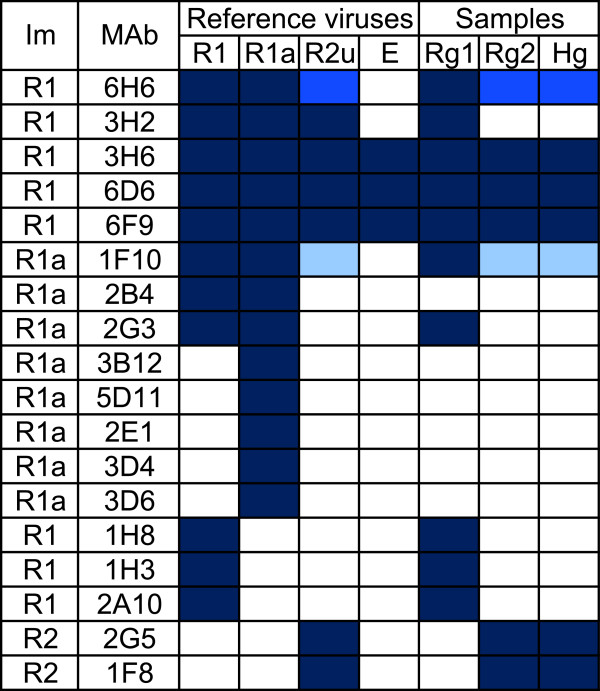
**Antigenic characterization using specific MAb panels of the rabbit hemorrhagic disease viruses (RHDVs) identified in Sardinia since 2008.** MAb reactivity is proportional to the depth of blue. Dark blue: > 80%; medium blue: 40-80%; light blue: 10-40%; white: no reactivity. Im: RHDV used as immunogen in MAb production; R1: RHDVBs89 – RHDV used as reference; R1a: RHDVPv96 – RHDVa subtype used as reference; R2: RHDV2_10-32. Reference viruses used in the ELISAs. R1: RHDVBs89; R1a: RHDVPv96; R2u: RHDV2Ud11; E: EBHSVBs91 – EBHSV used as reference. Groups of RHDV collected in Sardinia. Rg1: RHDV identified in rabbits from 2008 to May 2011; Rg2: RHDV identified in rabbits after October 2011; Hg: Cape hares affected by a RHD-like disease collected in December 2011.

**Figure 3:**
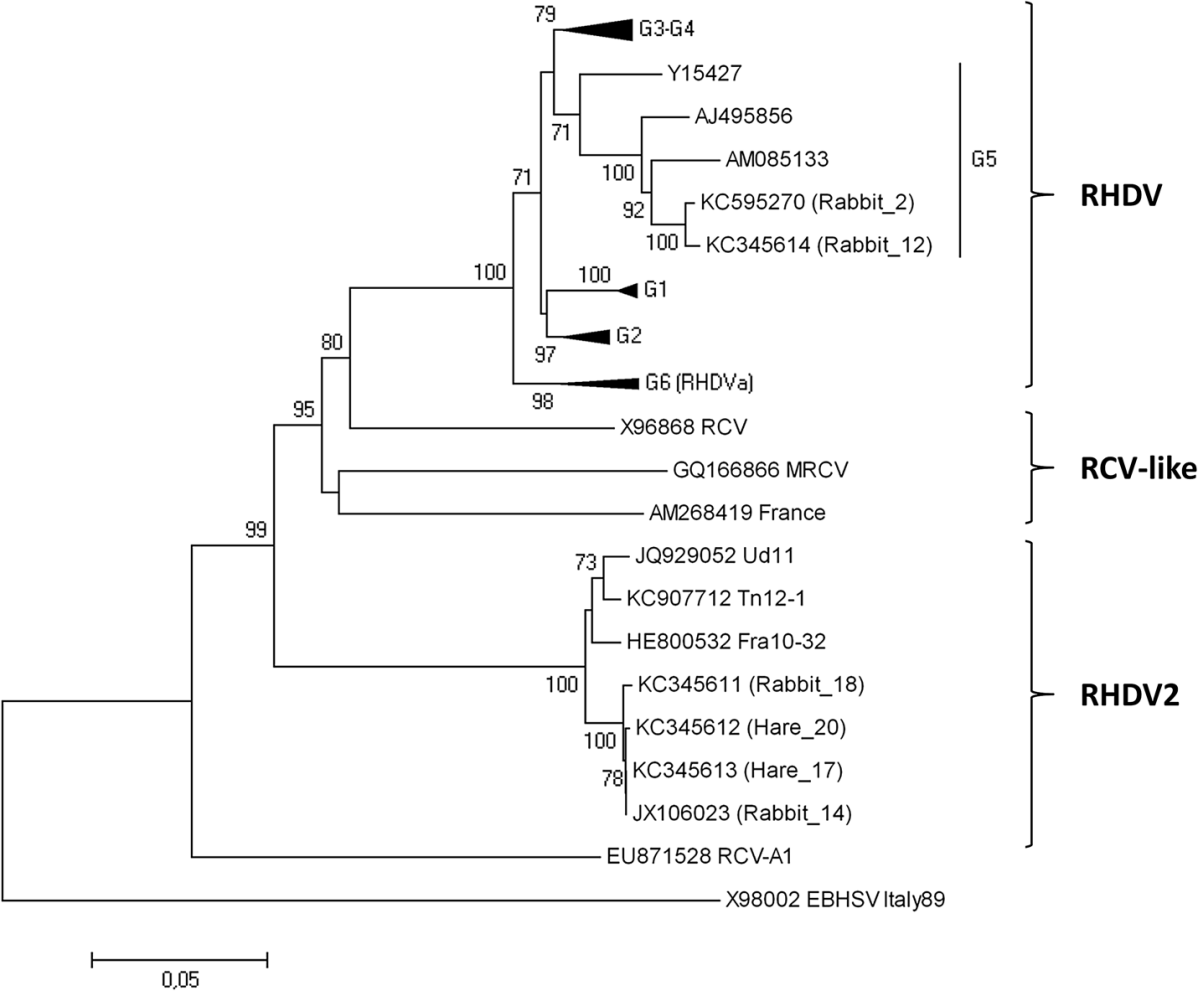
**Phylogenetic analysis of rabbit hemorrhagic disease virus (RHDV) isolates and RHDV2 isolates identified in Italian hares and rabbits.** For the phylogenetic analysis of the VP60 sequences, the neighbor-joining method was applied based on the Kimura 2-parameters using the software package MEGA5 [20]. The *European brown hare syndrome virus* (EBHSV) strain BS89 (GenBank accession number: X98002) was used as an outgroup to root the tree. Bootstrap probability values above 70% for 1000 replicate runs are indicated at the nodes. Twenty selected sequences representing previously described RHDV genogroups (G1-G6, [18]), four sequences of RCV-like isolates, and the sequences of the RHDV2 isolates identified in France in 2010 (strain 10–32) and in Italy (strains Ud11 and Tn12-1) were included. The GenBank accession numbers of these strains are indicated. The RHDV sequences from rabbits isolated before spring 2011 (accession numbers: KC595270 and KC345614) belong to genogroup 5, whereas those from rabbits and hares collected after this period (accession numbers: KC345611, KC345612, KC345613, and JX106023) belong to the genogroup RHDV2.

At the end of 2011, the National Laboratory and the World Organization for Animal Health (OIE) Reference Laboratory for RHD received six rabbit and seven Cape hare liver samples, which were collected from dead animals between April and December of 2011 in different parts of Sardinia, for RHD and EBHS diagnostic confirmation and virus typing (Figure 1; Table 1). At necropsy, performed at the Istituto Zooprofilattico Sperimentale of Sardinia (IZSS), all rabbits displayed the main classical RHD lesions [10,15], a pale, swollen and friable liver, a dark black and enlarged spleen, and petechial hemorrhages on the lungs and kidneys. The trachea presented hemorrhages and a foamy bloody mucous. Additionally, enteritis of the small intestine was noted. Histologically, the liver displayed the most significant lesions with multifocal coagulative necrosis, steatosis and a modest periportal mononuclear infiltrate. The antigenic profile of the isolates was assessed using anti-RHDV monoclonal antibodies (MAb) and a sandwich ELISA as previously described [11,16,17] with the addition of two recently produced MAbs specific for RHDV2. The results of the antigenic characterization of the six rabbit liver extracts are summarized in Figure 2. Additional RHDV-positive rabbit samples collected in Sardinia, i.e. ten samples from 2008 to spring 2011 and one sample in early 2012, are included in Figure 2. The reactivity patterns of the RHDV samples collected prior to May 2011 (Rg1) were very similar to that of the reference RHDVBs89 (R1) isolate, except for MAb 2B4, which was unreactive with all isolates. However, the isolates collected from October 2011 (Rg2) showed an RHDV2 (R2u) profile. They were unreactive with the MAbs specific for RHDVBs89 (R1) and RHDVa (R1a), but they were reactive with some of the cross-reactive MAbs (3H6, 6D6 and 6F9) and with the two new MAbs specific for RHDV2 (2G5 and 1F8). The genetic study of the isolates was performed by sequencing the VP60 gene of members of the two antigenically distinct rabbit groups as previously described [11]. The phylogenic results, reported in Figure 3, clearly show that the genomic profiles of the RHDV strains identified in rabbits sampled before spring 2011 belong to the original RHDV, particularly to the genogroup 5 [18]. However, the genomic profiles of RHDV strains identified in rabbit samples collected after October 2011 are highly related to the first RHDV2 strains identified in France and Italy [10-12] with an average nucleotide identity at VP60 of 97.8% and an amino acid identity close to 99%. Seven livers of Cape hares (*Lepus capensis*) (Table 1) were also received, of which six came from wild animals and one from a captive hare farm. All tested positive for lagovirus in the diagnostic ELISA performed at IZSS. Lesions indicative of EBHS with a pale discoloration of the liver, splenomegaly and congestion of the other parenchymal organs were observed during the necropsy [15]. Histologically, the liver showed massive necrosis involving the whole lobule with moderate mononuclear inflammatory infiltrate and little fatty degeneration. In all the hare livers, we confirmed the initial laboratory results that the lagovirus was present but, surprisingly, we again identified RHDV2 and not EBHSV. This conclusion was drawn from both the antigenic profile of the virus identified in the Cape hare group, which corresponded to RHDV2Ud11 (Hg line in Figure 2), and the high sequence correlation with the VP60 gene of RHDV2 (Figure 3). In fact, the amino acid sequences of the viral VP60 gene in rabbit number 14 and hare number 17 were identical. Interestingly, these animals were found dead at the same location (Sardara) within a few days of each other. Since RHDV2 isolates were able to hemagglutinate the human red cell (HA) of group “0” [11], we performed such a test on three hare and three rabbit RHDV2 positive livers. HA was performed using 1% of red blood cells (RBC) in PBS pH 7.2 at 4 °C [4]. Samples were 10% w/v liver extracts in PBS. All samples tested HA positive with titers ranging between 1/1280 and 1/10240. This result, coupled with that obtained by ELISA, demonstrated a similar viral load in the liver of the hares and rabbits tested. The registered titers are the same, on average, as those reported in cases of infection by classical RHDV [4,11]. Altogether, the data revealed that the epidemic that affected rabbits and Cape hare in Sardinia during the autumn/winter 2011 was due to just one virus identified as RHDV2. In light of this unexpected result, we performed genetic analyses, to confirm the identity of the host species on all the lagomorphs identified as Cape hares during necropsies. The genetic identification of the species was performed by comparing sequences of cytochrome b (cyt b) and cytochrome c oxidase I (COI) mitochondrial genes [19]. Sequence similarity was found using Genbank BLAST and the phylogenetic tree was constructed by the neighbor-joining method, generated by Kimura’s two-parameter model on MEGA 5.1 software [20] (data not show). The cyt b sequences were most similar to *Lepus capensis mediterraneus* and two similarity groups formed (Query coverage 100%, E value 0.0, Max identity 99% and Query coverage 100%; E value 2e-171; Max identity 98%). However, all of the sequences clustered with GenBank’s *Lepus capensis mediterraneus* sequences, but they were significantly (bootstrap = 100) separated from the sequences of *Lepus europaeus*, *Lepus capensis*, and *Lepus comus*. At present, there are no *Lepus capensis mediterraneus* COI gene sequences available in GenBank. However, the sequences obtained clustered and were significantly (bootstrap = 100) separated from those of *Lepus europaeus*, *Lepus capensis*, and *Lepus comus*. Thus, the genetic analyses confirmed that the RHDV2 positive lagomorphs were not rabbits but Sardinian Cape hares.

**Table 1:** Cases of rabbit hemorrhagic disease (RHD) recorded by IZS Sassari in rabbits and Cape hares in Sardinia from 2008 to 2012.

**N**	**Species**	**Type**	**Date**	**Place**	**Virus**
1	Rabbit	Rural farm	28/10/2008	TERRALBA	RHDV
2	“	“	19/11/2008	ULA TIRSO	“
3	“	“	10/02/2009	QUARTU S'ELENA	“
4	“	“	02/03/2009	SESTU	“
5	“	“	03/06/2009	DOLIANOVA	“
6	“	“	09/06/2009	DOLIANOVA	“
7	“	“	04/05/2010	NUORO	“
8	“	“	20/05/2010	BITTI	“
9	“	“	22/09/2010	VILLAMASSARGIA	“
10	“	“	16/06/2010	NUORO	“
11	“	“	28/04/2011	SESTU	“
12	“	“	12/05/2011	SINNAI	“
13	“	“	05/10/2011	SINNAI	RHDV2
14	“	Wild	30/11/2011	SARDARA	“
15	Hare	“	01/12/2011	PAULILATINO	“
16	Rabbit	“	02/12/2011	CAGLIARI	“
17	Hare	“	02/12/2011	SARDARA	“
18	Rabbit	“	08/12/2011	ALGHERO	“
19	Hare	“	09/12/2011	TRESNURAGHES	“
20	“	Captive	12/12/2011	LAS PLASSAS	“
21	“	Wild	20/12/2011	NORAGUGUME	“
22	“	“	23/12/2011	SARDARA	“
23	“	“	27/12/2011	OLIENA	“
24	Rabbit	“	28/03/2012	DOLIANOVA	“

## Discussion

The present work reports the occurrence in Sardinia of an RHDV2 epidemic that affected rabbits and Cape hares (*Lepus capensis mediterraneus*), causing in both species a similar necrotizing hepatitis with a comparable degree of mortality. All the affected animals were wild or were from small rural farms; therefore, the collected epidemiological data were not enough for an acceptable estimate of the mortality rate. However, the simultaneous occurrence of the epidemic in both species and the high genetic correlation between the viral strains isolated in rabbits and hares, 100% VP60 amino acid identity in the case of simultaneously sampled isolates in Sardara, demonstrate the uniqueness of the etiological agent. Thus, this is the first report of a lagovirus with a host range that includes both the *Oryctolagus cuniculus* and a *Lepus* species. This conclusion is also supported by the available epidemiological data previously collected in Sardinia. Indeed, all the hare liver samples, which were all from Cape hares, conferred to the IZSS over the past 20 years, were negative for lagovirus in spite of the endemic presence of RHDV. This unequivocally demonstrates that Cape hares, as others *Lepus* species, are not susceptible to RHD. However, only seroepidemiologic surveys and/or experimental infections, will demonstrate whether Cape hares are also not susceptible to a non-pathogenic RHDV infection.

On the contrary, the present available epidemiological data strongly suggests that the European brown hare (*Lepus europaeus*) is not, or is consistently less, susceptible to RHDV2. The brown hare is the most relevant European *Lepus* species, being prevalent in central Europe, France and continental Italy but not in the main Italian islands, Sardinia and Sicily, where it is not a native species and regional laws forbid its introduction. It is the subject of intense surveillance plans due both to its importance as game for hunters and as the host of EBHSV. For example, at the end of 2010 an overt EBHSV epidemic was registered in France by the French Wildlife Health Surveillance (SAGIR) network [21] in an area where RHDV2 was prevalent. However, the laboratory results always showed EBHSV in the hare livers, never RHDV2. Similarly, a second large outbreak in rural farm and wild rabbits caused by RHDV2 (Tn12-1) [12] was registered in northeastern Italy in areas where there are high-density populations of brown hares, but again only EBHSV was identified. Therefore, the data available allow us to propose that the brown hare is not susceptible to the RHD-like disease caused by RHDV2. Again, only specific seroepidemiologic studies and/or experimental infections will show if the brown hare is really not susceptible to RHDV2 also as a non-pathogenic infection.

EBHSV has never been reported in *Lepus capensis* but we cannot conclude that the Cape hares are not susceptible to it. In fact, this could be simply due to the lack of the virus introduction onto the island and/or to the absence of brown hares. In this context, it is interesting to note that some degree of genetic susceptibility among *Lepus* species towards EBHSV has already been observed. In Sweden, two hare species, *Lepus europaeus* and *Lepus timidus*, coexist in some parts of the country [22]. EBHSV causes disease in both species, but it has a higher incidence in the brown hare. In addition, the disease rarely occurs in the northern regions of Sweden where only *Lepus timidus* is present. This evidence supports the hypothesis that the main host of EBHSV is the brown hare, whose presence is necessary to keep the level of viral infectivity sufficiently high in the field to allow a continuous diffusion to *Lepus timidus*[22].

The high susceptibility to RHDV2 versus the null susceptibility to RHDV of Cape hares indicates that the genetic barrier to lagovirus among lagomorphs is controlled by few but still largely unknown factors. At present, the only candidate is the histo-blood group antigens (HBGA) expressed on the intestinal surface of rabbits and to which RHDV specifically binds [23,24]. Interestingly, RHDV2 hemagglutinate RBC type “0”, which expresses H type 2 antigen on its surface [24], as efficiently as the majority of the RHDV isolates and differently than the EBHSV isolates that have a much lower HA efficiency [22]. This suggests two considerations. Firstly, although both RHDV and RHDV2 hemagglutinate RBC type “0”, the overt difference in susceptibility of Cape hares to the two RHDV viruses strongly indicates that they infect rabbits through two distinct genetic factors, or, more probably, a genetic variant of the same factor, such as a different HBGA or patterns of HBGA [24]. This could also explain the large difference in the mortality rates obtained in the experimental infections of rabbits with the two viruses [11] and the field observation that rabbits less than 6 to 7 weeks old, which are not susceptible to RHD, develop the disease when infected by RHDV2 [10,14]. Secondly, there are likely differences between the HBGA of the Cape hare and brown hare and these differences could have been further increased by the selection induced in brown hares by EBHSV during more than 30 years of coevolution. Indeed, brown hare populations could be enriched for particular HBGA alleles whose presence induces an increased resistance to EBHSV [24].

Moreover, a secondary non-genetic factor responsible for the observed different susceptibility may be the immunological status of the brown hare and the Cape hare in respect to lagoviruses at the population level. Extended serological surveys showed that a large percentage of the brown hare populations tested positive for antibodies to EBHSV [25]. Otherwise, the serological data on lagoviruses are not available for Cape hares, but, considering the absence of EBHSV in Sardinia, it is likely that they are serologically negative. Therefore, this different immunological status could be a contributing factor that makes the brown hare less susceptible than the Cape hare to RHDV2 infection. Indeed, even if the antigenic difference between RHDV2 and EBHSV is relevant, it has been shown for lagoviruses that some degree of protection from the disease can occur although there is a limited antigenic relationship between the viruses [26].

The identification and diffusion of RHDV2 in Spain after the spring of 2011 [13,14] will throw further light on the relationship between RHDV2 and the different *Lepus* species. Indeed, on the Iberian Peninsula, in addition to wild rabbit populations and the brown hare in the Pyrenees region, there are two other *Lepus* species, *Lepus granatensis*, which is present in a large part of Spain, and *Lepus castroviejoi*, which is present only in a limited area in the north of the country [27]. Interestingly, *Lepus granatensis* seems to be the species genetically closer to *Lepus capensis*[27,28] and, therefore, it has a concrete possibility of being susceptible to RHDV2. In Spain, similar to Sardinia, there have been no reports of EBHS in hares so far. In addition, it will be interesting to study the diffusion of RHDV2 in the southern regions of Italy, where RHDV2 was recently identified in rabbits (A. Guercio and A. Camarda, personal communication), considering the presence of residual populations of the native species *Lepus corsicanus*.

In conclusion, the demonstration that the Sardinian Cape hare is a host for RHDV2 adds a further phenotypic feature to this lagovirus that is distinctive from RHDV, whose unique host is the European rabbit (*Oryctolagus cuniculus*). The emergence of new viral disease is inherent to the continuous evolution of viruses, a very complex biological issue [29]. The emergence of RHD and the origin of RHDV, in spite of the available data, are still at the level of speculations [3]. Le Gall-Reculé et al. [11], considering the genetic and epidemiological data available on RHDV2 and with background knowledge on RHDV, conclude that the emergence of RHDV2 could be due to the jump of an unknown lagovirus into the rabbit populations. Our finding that RHDV2 causes a similar disease in a second lagomorph species and because the level of fitness of RHDV2 for Cape hares seems very similar to that of rabbits, in that the virus already seems rather adapted to the new host, reinforces this hypothesis [29]. This could be demonstrated by searching for non-pathogenic lagoviruses in the lagomorph species living in the wild, first of all in France, using the same strategy and methods employed to discover non-pathogenic RCV [6,8]. De facto, France is the first and main candidate for the geographical place where the jump could have occurred [10,11]. This is not only because RHDV2 was initially identified there and rapidly diffused from there, but also because previous phylodynamic studies on RHDV indicated that France has been the most relevant source population for the virus [3].

Finally, this new data are also epidemiologically relevant, indicating the urgency of increased hare species’ surveillance plans to quickly detect the eventual presence of RHDV2 as the cause of this new disease.

## Competing interests

The authors declare that they have no competing interests.

## Authors’ contributions

All the authors contributed to the general interpretation of the data and the writing of the manuscript. In addition, GP performed the initial diagnostic ELISA and collected epidemiological data. PC carried out the molecular phylogenetic studies of the viral isolates. CM carried out the molecular genetic studies for the species confirmation of lagomorphs involved in the study. RS and CL performed necropsies on the animals and the anatomohistopathological examinations. GB carried out the immunoassay for virus typing. GL supplied the original French RHDV2 sequence and the French epidemiological data. AL participated in the interpretation of the epidemiological and diagnostic data and in drafting the manuscript. LC designed the antigenic and HA experiments and drafted the manuscript. All authors read and approved the final manuscript.
